# Model parameters influencing the cost-effectiveness of sacubitril/valsartan in heart failure: evidence from a systematic literature review

**DOI:** 10.1007/s10198-022-01485-3

**Published:** 2022-07-05

**Authors:** Clare Proudfoot, Raju Gautam, Joaquim Cristino, Rumjhum Agrawal, Lalit Thakur, Keith Tolley

**Affiliations:** 1 Novartis Pharma AG, Basel, Switzerland; 2 Novartis Healthcare Pvt. Ltd., Hyderabad, India; 3 Novartis Pharmaceuticals Corporation, East Hanover, NJ USA; 4 Tolley Health Economics Ltd., Unit 5, 11-13 Eagle Parade, Buxton, SK17 6EQ Derbyshire UK

**Keywords:** Cost-effectiveness, Economic model, Heat failure, Sacubitril/valsartan, Sensitivity analysis, C00

## Abstract

**Objectives:**

To summarize cost-effectiveness (CE) evidence of sacubitril/valsartan for the treatment of heart failure (HF) patients with reduced ejection fraction (HFrEF). The impact of different modeling approaches and parameters on the CE results is also described.

**Methods:**

We conducted a systematic literature review using multiple databases: Embase^®^; MEDLINE^®^; MEDLINE^®^-In Process; NIHR CRD database including DARE, NHS EED, and HTA databases; and the Cost Effectiveness Analysis registry. We also reviewed HTA countries’ websites to identify CE reports of sacubitril/valsartan, published up to 25-July-2021. Articles published in English as full-texts, conference-abstracts, or HTA reports were included.

**Results:**

We included 44 CE models [39 from 37 publications (22 full-texts; 15 conference-abstracts) and 5 HTAs; Europe, *n* = 20; North and South Americas, *n* = 14; Asia and Australia, *n* = 10]. Most models adopted a Markov structure with constant transition probabilities of events (*n* = 27) or a mix of Markov and regression-based models (*n* = 16), with variations in structural assumptions and chosen parameters. Study authors concluded sacubitril/valsartan to be a cost-effective therapy in 37/41 models in chronic HFrEF patients and 2/3 models in hospitalized patients stabilized after an acute decompensation for HF. CE models showing sacubitril/valsartan not to be a cost-effective treatment generally modeled a shorter time horizon. Effect of sacubitril/valsartan on cardiovascular and all-cause mortality, cost, duration of effect and time horizon was the main model drivers.

**Conclusions:**

Most evidence indicated sacubitril/valsartan is cost-effective in HFrEF. The use of a lifetime horizon is recommended in future models as HF is a chronic disease. Data on the CE of sacubitril/valsartan in the inpatient setting were limited and further research is warranted.

**Supplementary Information:**

The online version contains supplementary material available at 10.1007/s10198-022-01485-3.

## Introduction

The health technology assessment (HTA) of new drugs normally involves the development of cost-effectiveness (CE) models, which generally extrapolate the costs and health outcomes associated with the use of new drugs/technologies over the lifetime of patients. A CE model involves two important components: structural aspects of the model (i.e., health states or the events) and input parameters [i.e., comparator, treatment effect (TE), health utilities, costs, and resource use]. The assumptions made around the chosen structural aspects and input parameters can impact the results from a CE model and consequently the healthcare decision making [1–3].

Recently, there has been a growing interest in understanding the influence of choices around model structure and parameters on the results from the model [4–6]. Frederix et al. showed that differences in the choice of parameters in CE analyses comparing tamoxifen and anastrozole for the treatment of breast cancer were associated with major differences in CE results despite using the same clinical trial for TEs [4]. Hlatky et al. observed variations in incremental CE ratios (ICERs) per quality-adjusted life-year (QALY) gained when assuming the convergent survival curves versus parallel survival curves [United States (US)$91,500 vs US$44,900] in the economic analysis of implantable cardioverter defibrillators versus drug therapy in patients with cardiac arrhythmia [7]. The effect of structural assumptions and input parameters on CE results are explored to a greater/lesser extent by model developers in sensitivity analysis (SA) or during the model review, for example, by the Evidence Review Group (ERG) of the National Institute for Health and Care Excellence (NICE) or by peer review during publication.

Sacubitril/valsartan (Entresto^®^), a novel, first-in-class angiotensin-receptor neprilysin inhibitor (ARNI) was approved in 2015 for the treatment of patients with chronic heart failure (HF) with reduced ejection fraction (HFrEF) [8, 9]. The PARADIGM-HF trial demonstrated a significant reduction in all-cause and cardiovascular mortality and HF hospitalization with sacubitril/valsartan versus enalapril in patients with HFrEF [10]. The PIONEER-HF trial demonstrated in-hospital initiation of sacubitril/valsartan to be safe and to be associated with a significant reduction in N-terminal pro-B-type natriuretic peptide (NT-proBNP) levels and HF rehospitalizations compared with enalapril [11, 12]. Recognizing the clinical benefit of sacubitril/valsartan, particularly in patients with sub-normal ejection fraction as shown in the PARAGON-HF trial [13], the Food and Drug Administration of the US recently approved an expanded indication for sacubitril/valsartan in chronic HF [14]. The European Society of Cardiology guidelines recommend substitution of angiotensin-converting enzyme inhibitor (ACEi) or angiotensin-receptor blocker (ARB) with ARNI in appropriate HFrEF patients [15]. The American College of Cardiology Expert Consensus Decision Pathway for Optimization of Heart Failure Treatment committee in its 2021 update recommends that in patients with new onset of HFrEF, the treatment should be started with either an ARNI/ACEi/ARB or a β-blocker, and that ARNI is preferred over ACEi/ARB [16].

Several publications and HTA reports have been published on the CE results of sacubitril/valsartan since its approval for HFrEF [17–21]. A recently published systematic literature review (SLR) briefly summarized the CE analyses and results of sacubitril/valsartan in HFrEF [22]. However, the SLR lacked a systematic assessment of the model structures and impact of parameters on the CE results of sacubitril/valsartan. In this study, we reviewed the literature systematically and explored the impact of different modeling approaches and parameters on the CE results for sacubitril/valsartan.

## Methods

### Data sources and searches

This SLR was conducted following the Preferred Reporting Items for Systematic Reviews and Meta-Analysis (PRISMA) guidelines [23]. Systematic searches were conducted using the Embase.com platform, which included Embase and MEDLINE databases from inception to July 25, 2021. The MEDLINE Epub ahead of print, in-process, and other non-indexed citations were searched using PubMed. Supplementary Tables S1 and S2 provide details of the search strategy, which included both Medical Subject Headings and free-text words for disease conditions (heart failure, cardiac failure), sacubitril/valsartan, economic analysis, and model.

We also searched the National Institute for Health Research Centre for Reviews and Dissemination database comprising the Database of Abstracts of Reviews of Effects, the National Health Services Economic Evaluation Database, and the HTA database. The Cost Effectiveness Analysis Registry (https://research.tuftsnemc.org/cear4/Default.aspx) was searched to identify any additional publications. We searched the websites of the following HTA agencies to identify manufacturer submissions or ERG reports: UK NICE, Scottish Medicines Consortium (SMC), US Institute for Clinical and Economic Review (ICER), Australian Pharmaceutical Benefits Advisory Committee (PBAC), and Canadian Agency for Drugs and Technologies in Health (CADTH). A bibliographic search of relevant reviews was also performed to identify additional publications. Only articles in English were included.

### Study selection

Supplementary Table S3 presents details of the inclusion and exclusion criteria. Scientific articles published as full-text publications or conference abstracts and reports of HTA agencies were included if they reported the CE results of sacubitril/valsartan in patients with chronic HF. Two independent reviewers (R.G. and L.T.) screened all retrieved citations based on title and abstract per predefined eligibility criteria; a third independent reviewer (R.A.) resolved discrepancies by consensus after a discussion. Full-text publications were obtained and screened, and those satisfying the inclusion criteria were included for data extraction. Multiple publications from the same study were linked.

### Data extraction and quality assessment

Data extraction of the included publications and HTA reports was performed by one reviewer (R.G. or L.T., depending on the specific study). Quality checks of data were performed by the second reviewer (L.T. or R.G.) and differences were reconciled by the third reviewer (R.A.). Data on the structural assumptions, health states, model parameters, CE results, and sensitivity and scenario analyses were extracted into an extraction grid in Microsoft Excel. The methodological quality of full-text publications was assessed using the Drummond and Jefferson checklist for economic evaluations [24].

### Analysis

We analyzed the data on model structure, assumptions, CE results in the base-case, and the parameters considered in SAs. Parameter results from Tornado diagrams, where available, were extracted. The parameter at the top of the Tornado diagram was considered to have the greatest influence on CE results. The parameters described in the study results/conclusions to have an impact on the CE results were also analyzed. Data are summarized using descriptive statistics (i.e., numbers and/or percentages).

## Results

**Fig. 1:**
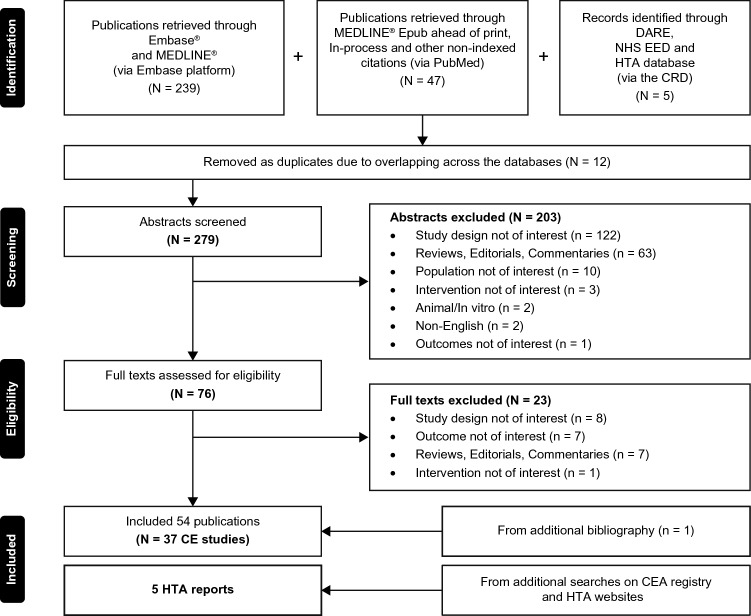
PRISMA diagram. *CE* cost-effectiveness, *CEA* cost-effectiveness analysis, *CRD* Center for Reviews and Dissemination database, *DARE* Database of Abstracts of Reviews of Effects, *EED* Economic Evaluation Database, *HTA* health technology assessment, *NHS* National Health Services, *PRISMA* Preferred Reporting System for Systematic Reviews and Meta-Analysis

The literature search provided 291 citations in total. After screening the titles and abstracts and then the full-text manuscripts, 53 publications were included. One additional publication was identified from the bibliographic search. After linking multiple publications, 37 studies [18–21, 25–57] were included. Additional searches provided five reports produced by the HTA agencies [17, 58–61] (Fig. 1).

### Summary of studies

A total of 22 full-text publications, 15 conference abstracts, and 5 HTA reports (1 each from the NICE [17], US ICER [61], CADTH [58], PBAC [60] and SMC [59]) were included. Of these, seven were from the US [18, 20, 30, 33, 35, 52, 61], three from Canada [51, 55, 58], three from Australia [27, 56, 60], and two each from the UK [17, 41], Germany [32, 48], Italy [29, 50], the Netherlands [21, 43], Russia [53, 57], and Singapore [19, 40]. Seventeen of the included studies were published by academic research institutions. While most studies modeled populations with chronic HFrEF in the outpatient setting, three studies modeled patients stabilized in hospital after an acute decompensation for HF [33, 56, 57] (﻿Supplementary Table S4).

### CE models of sacubitril/valsartan in chronic HFrEF in outpatient setting

#### Model structure and outcomes

Forty-one CE models (36 from 34 publications and 5 from HTAs) were identified for patients with chronic HFrEF in outpatient setting. Of these, 17 were separate/distinct models, whereas 21 were country adaptations of the same overall base model. In three models, it was unclear as these were published as conference abstracts. Notably, there were two types of model: (1) Markov structure modeling the constant transition probabilities of the events (i.e., HF hospitalization, all-cause and/or cardiovascular mortality) considered in the health states during the cycle length of the model (24 models), and (2) Markov structure with regression-based models using a series of regressions to predict the outcomes over the lifetime horizon based on patient characteristics and treatment received (16 models). Markov models using constant transition probabilities varied in structure and health states. A study from the US modeled that patients with HF each month had a risk of either surviving without further complication, becoming hospitalized, or dying [18]. Another model from the US assumed that patients had a monthly risk of HF hospitalization, non-HF hospitalization, emergency department (ED) visit for HF, treatment intolerance, and cardiovascular or non-cardiovascular death [20]. Several models were also based on HF patients transitioning between the four New York Heart Association (NYHA) functional classes (I–IV) and death [19, 35, 49, 51, 52]. A simpler structure based on two health states, “alive” and “dead”, was also commonly used across models. Other health states included in models were “stable HF”, “hospitalization due to worsening HF” and “death” [18, 26, 29, 36, 50]; “stable HF”, “ward hospitalization”, “intensive care unit hospitalization”, and “death” [21, 48]; and “alive with HF”, “hospitalization (no readmission, 30-day readmission)”, “cardiovascular death”, and “non-cardiovascular death” [36]. The assumptions considered under the “alive” health state also varied greatly, e.g., “alive with HF”, “HF without complication”, “hospitalization”, “ED visit”, “alive with adverse events (AEs)”, “hypotensive AE”, “outpatient treated HF”, and “HF patients receiving home care only”.

CE models using a Markov structure with regression-based modeling generally considered hospitalizations, AEs, and health-related quality of life (HRQoL) within the “alive” health state. A model based on Markov structure using regression equations for outcomes was developed by McMurray et al. for the UK [41] and was adapted for several countries, including Spain [37], Switzerland [25], Netherlands [43], Denmark [41], Sweden [28], Greece [47], Czech Republic [46], Portugal [26], Turkey [45], Singapore [40], Taiwan [31], South Korea [42], Brazil [39], Cost Rica [38], and Colombia [41]. A study from India by Gokhale et al. adopted a cost-consequence analysis to predict HF hospitalizations and mortality with sacubitril/valsartan versus ACEi in patients with chronic HFrEF [54].

The mean age of patients in these models ranged from 60 [35, 36] to 75 years [43], with the majority of patients being male [25, 28, 29, 41, 43, 45]. Among models that reported information on the NYHA functional class [19, 20, 25, 28, 29, 32, 35, 42, 43, 50–52], the majority of patients were in NYHA class II. Most models used enalapril as the comparator, cost-utility analysis as the economic analysis type, payer’s perspective, lifetime horizon, and a monthly cycle. The discount rates on costs and health outcomes ranged from 1.5 [21, 43] to 5% [38, 39, 41, 42, 48]. The model results were commonly reported as total costs, ICERs, life-years gained, QALYs gained, and willingness-to-pay (WTP) thresholds. Despite using different model structures, health states, and parameters, the authors concluded sacubitril/valsartan to be a cost-effective therapy compared with an ACEi, ARB or placebo in the base-case results of all except four models (two from the US [30, 52], one from Singapore [19], and one from Thailand [36]), with ICERs per QALY gained being below the country-specific WTP thresholds (US, US$50,000; Canada, CA$50,000; UK, ₤20,000; Germany/Spain, €30,000; Italy, €40,000; Switzerland, CHF50,000). CE models that showed sacubitril/valsartan not to be a cost-effective treatment option generally had different assumptions from those indicating CE, particularly relating to time horizon. In the study using cost-consequence analysis, the authors demonstrated sacubitril/valsartan to be associated with substantial cost-savings [54] (Supplementary Table S4).

#### Sensitivity analyses

Thirty-seven models provided information on SA, which included deterministic SA or probabilistic SA (PSA). One-way SA was performed in 33 models, with parameter details provided in 29 models and Tornado diagram in 24 models only. Supplementary Table S4 lists the details on one-way SA parameters. Two-way SA was reported in 5 models [20, 32, 35, 49, 52] and PSA in 31 models. In one-way SA, 17 models used 95% confidence intervals (CIs) where applicable, whereas, in others, the parameters varied over plausible ranges (10 models) or specified very large variations (from ± 5% up to ± 50%). Fifteen models used 10,000 iterations and 9 used 1,000 iterations, with Monte Carlo simulation performed in 17 CE analyses. Different parametric distributions were used in PSA for various parameters: for baseline risk, beta (*n* = 10), multivariate normal (*n* = 3), and normal/lognormal (*n* = 2); for TE or relative risk (RR), lognormal (*n* = 12), beta (n = 3), and triangular (*n* = 1); for utility values, beta (*n* = 12), multivariate normal (*n* = 1), and triangular (*n* = 1); and for costs, gamma (*n* = 10), lognormal (*n* = 3), normal (*n* = 1), and triangular (*n* = 1). Gompertz distribution was used to estimate the baseline risk in 14 models, whereas Weibull (*n* = 8) or exponential (*n* = 6) distributions were explored in alternative scenarios.

#### Overview of model parameters

A wide range of parameters was used in the sensitivity/scenario analyses in the CE models of sacubitril/valsartan (Supplementary Table S4). Of note, time horizon, cost of sacubitril/valsartan, cost of HF hospitalization, utility increment with sacubitril/valsartan, cost of comparator, and TE on HF hospitalization were the most common parameters used in Markov models with constant transition probabilities (Supplementary Fig. 1A). In the models using a mix of Markov and regression models, the coefficient of constant for cardiovascular mortality, coefficient of sacubitril/valsartan for utility, coefficient of constant for hospitalization, coefficient of age for hospitalization, coefficient of sacubitril/valsartan for hospitalization, coefficient of age for cardiovascular mortality, and coefficient of sacubitril/valsartan for cardiovascular mortality were the frequently used parameters (Supplementary Fig. 1B).

#### Parameters with the highest impact on CE results

**Fig. 2:**
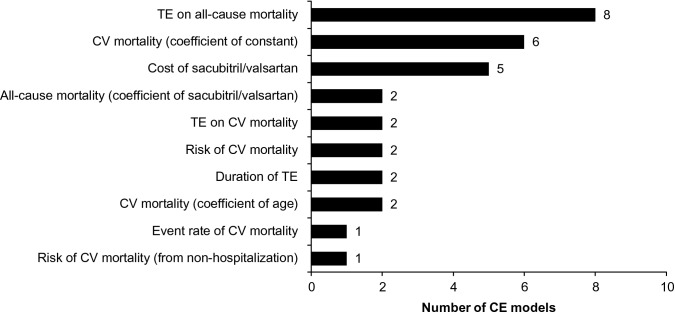
Model parameters with largest impact on ICERs (top parameter in Tornado diagram) in cost-effectiveness models of sacubitril/valsartan. *CV* cardiovascular, *ICER* incremental cost-effectiveness ratio, *TE* treatment effect

We observed that baseline risk or TE on cardiovascular mortality (14 models), all-cause mortality (10 models), cost of sacubitril/valsartan (5 models), and duration of TE (2 models) were parameters with the greatest impact on ICER values (i.e., top parameter in the Tornado diagram) and key drivers of the sacubitril/valsartan CE models (Fig. 2). Time horizon was also reported to influence the results. Time horizon was not shown in the Tornado diagrams but was considered in scenario analyses in many studies. These parameters are discussed below in more detail.

##### Cardiovascular and all-cause mortality

Fourteen CE analyses showed cardiovascular mortality as the key driver of the sacubitril/valsartan model (Table 1) [19, 26, 27, 29, 31, 35, 36, 38–41, 43, 60]. A study from Thailand concluded that sacubitril/valsartan may not be a cost-effective therapy in the base-case (ICER/QALY THB162,276; WTP THB160,000), and that risk of cardiovascular death in patients who received enalapril was the most influential model driver [36]. One-way SA indicated that the constant coefficient (Portugal [26], Singapore [40], Taiwan [31], Brazil [39], Costa Rica [38], and Colombia [41]) and age-squared coefficient (the Netherlands [43], Denmark [41]) in the Gompertz distribution of cardiovascular mortality were parameters that had the most influence on the CE results, but ICERs at the lower bound of the 95% CI remained below the WTP limits. In the CE models from Portugal (€36,059) [26], Denmark (Kr285,710) [41], Australia (A$71,404) [27], Singapore (SG$1,447,103) [19], and Thailand (THB290,000) [36], the ICERs estimated at the upper bound of the 95% CI were above the country-specific thresholds. This finding was not unexpected given that mortality is typically the key driver of CE analyses in cardiovascular disease.

**Table 1:** Studies with cardiovascular mortality or all-cause mortality as the key driver of sacubitril/valsartan and associated ICERs in base-case and sensitivity analyses

Study (Country)	Parameter description	Base-case		One-way sensitivity analysis			
		Value	ICER/QALY	Values		ICER/QALY	
				Lower 95% CI	Upper 95% CI	Lower 95% CI	Upper 95% CI
***Cardiovascular mortality as the cost-effectiveness driver of model***							
Borges et al. 2020 [26] (PT)	Coefficient of constant	− 12.665	€22,702	− 13.935	− 11.396	€14,570	€36,059
Chin et al. 2020 [27] (AU)	Incidence of CV death, enalapril	–	AU$40,513	–	–	AU$31,461	AU$76,100
McMurray et al. 2018 [41] (DK)	Coefficient of CV mortality: Age^2^	0.001	Kr174,000	0.000	0.001	Kr116,122	Kr285,710
Lacey et al. 2018 [38] (CR)	Coefficient of constant	− 12.665	₡6,108,752	− 13.934	− 11.395	₡5,068,070	₡8,581,518
McMurray et al. 2018 [41] (CO)	Coefficient of constant	− 12.665	COP$39.5 Mn	− 13.934	− 11.395	COP$24.1 Mn	COP$55.0 Mn
Lacey et al. 2018 [39] (BR)	Coefficient of constant	− 12.665	BRL28,154	− 13.934	− 11.395	BRL19,833	BRL47,674
Liang et al. 2018 [19] (SG)	CV mortality, HR	0.80	SG$74,592	0.62	1.00	SG$41,019	SG$1,447,103
*Lee et al. 2018 [40] (SG)	Coefficient of constant	–	–	–	–	–	–
Krittyaphong et al. 2018 [36] (TH)	CV mortality risk (from non-hospitalization), enalapril	0.0168	THB162,276	0.0150	0.0188	THB120,290	THB290,000
D’Angiolella et al. 2017 [29] (IT)	TE of sac/val on CV mortality	–	€19,487	–	–	–	–
Ramos et al. 2017 [43] (NL)	Coefficient of CV mortality: Age^2^	0.001	€17,600	0.000	0.001	€13,375	€33,393
*Fann et al. 2017 [31] (TW)	Coefficient of constant	–	–	–	–	–	–
King et al. 2016 [35] (US)	Risk of CV mortality, sac/val	–	US$50,959	–	–	US$23,657	Dominated
PBAC (HTA), 2016 [60] (AU)	CV mortality	–	< AU$45,000	–	–	–	–
***All-cause mortality***							
van der Pol et al. 2019 [48] (DE)	TE of sac/val on death, RR	0.84	€19,300	0.76	0.93	€11,000	€135,000
Zueger et al. 2018 [52] (US)	TE of sac/val on death, RR	0.84	US$143,891	0.76	0.93	US$112,000	US$225,000
McMurray et al. 2018 [41] (UK)	All-cause mortality- Gompertz (coef.): Sac/Val	− 0.161	£17,200	− 0.061	− 0.261	£12,700	£26,500
Gandjour and Ostwald 2018 [32] (DE)	TE of sac/val on death, HR	0.84	€26,278	0.74	0.94	€22,683	€38,466
Zaca 2018 [50] (IT)	TE of sac/val on death vs ICD, HR	1.02	–€98,500	0.82	1.26	Dominant	€55,000
Ademi et al. 2017 [25] (CH)	All-cause mortality (coef.): sac/val	− 0.161		− 0.061	− 0.261	CHF20,947	CHF38,104
van der Pol et al. 2017 [21] (NL)	TE of sac/val on death, RR	0.84	€19,113	0.76	0.93	€11,000	€45,000
Gaziano et al. 2016 [18] (US)	TE of sac/val on death, HR	0.84	US$45,017	0.76	0.93	US$35,357	US$75,301
NICE (HTA), 2016 [17] (UK)	All-cause mortality	–	£17,939	–	–	–	–
SMC (HTA), 2016 [59] (SC)	All-cause mortality	–	£18,348	–	–	–	£34,000

*AU* Australia, *BR* Brazil, *CH* Switzerland, *CI* confidence interval, *CO* Colombia, *CR* Costa Rica, *CV* cardiovascular, *DE* Germany, *DK* Denmark, *HR* hazard ratio, *HTA* health technology assessment, *ICER* incremental cost-effectiveness ratio, *ICD* implantable cardioverter-defibrillator, *IT* Italy, *NICE* National Institute for Health and Care Excellence, *NL* the Netherlands, *PBAC* Pharmaceutical Benefits Advisory Committee, *PT* Portugal, *QALY* quality-adjusted life-year, sac/val sacubitril/valsartan, *RR* relative risk, *SC* Scotland, *SG* Singapore, *SMC* Scottish Medicines Consortium, *TE* treatment effect, *TH* Thailand, *TW* Taiwan, *UK* United Kingdom, *US* United States

All-cause mortality was the model driver in 10 CE analyses (Table 1) [17, 18, 21, 25, 32, 41, 48, 50, 52, 59]. In one study from the US, which concluded that sacubitril/valsartan may not represent good value for money at a WTP of US$100,000 (i.e., ICER/QALY US$143,891), the TE of sacubitril/valsartan on mortality was the main value driver [52]. In this study, when the mortality hazard ratio (HR) was tested over the 95% CI, the ICERs/QALY gained ranged from US$112,000 to $225,000 [52]. In other models from the US [18], the UK [41], Italy [50], Switzerland [25], and the Netherlands [21], the ICERs obtained with the lower and upper bound of mortality HRs were below the country-specific thresholds. However, in three models (two from Germany [32, 48], one from the US [52]), the ICERs generated were higher than the specified marks at the upper bound of the 95% CI. While both studies from Germany used the same HR, the ICER/QALY at the upper bound of the 95% CI was €38,466 in one study (base-case €26,278) [32] and approximately €135,000 in another (base-case €19,300) [48]. The model design in both studies was different in terms of discounts on price of sacubitril/valsartan, indirect medical costs, and adjustment of PARADIGM-HF mortality rates based on Germany-specific data [32, 48].

##### Cost of sacubitril/valsartan

**Fig. 3:**
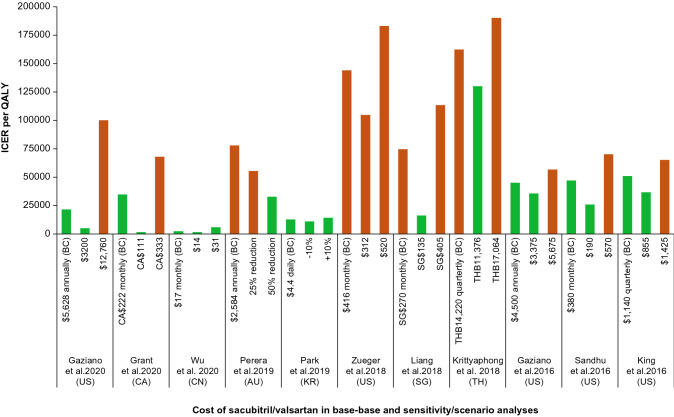
Studies of sacubitril/valsartan evaluating impact of cost of sacubitril/valsartan on ICERs in base-case and sensitivity/scenario analyses. The green and red bars indicate the ICERs below and above the country-specific WTP thresholds, respectively. The ICERs are presented in the currency of respective country as reported in the publication. *AU* Australia, *BC* base-case, *CA* Canada, *CN* China, *ICER* incremental cost-effectiveness ratio, *KR* South Korea, *QALY* quality-adjusted life-year, *SG* Singapore, *TH* Thailand, *US* United States

The cost of sacubitril/valsartan was a key influence on ICER in five models [33, 42, 49, 55, 56]. In the Canadian study, the de novo initiation strategy with sacubitril/valsartan (i.e., no preceding trial of ACEi/ARB) versus current care was shown to be cost-effective with an ICER/QALY of CA$34,727 at monthly cost of CA$222. When the monthly cost of sacubitril/valsartan was varied between CA$111 and CA$333, the resulting ICERs were CA$1,590 and CA$67,864, respectively [55]. Sacubitril/valsartan was shown to be a cost-effective therapy in China and South Korea even at the highest end of cost used in the analysis. Sacubitril/valsartan was concluded as not a cost-effective therapy in the base-case in Thailand; however, a 2% reduction in the daily cost of sacubitril/valsartan brought the ICER below the WTP limit (Fig. 3) [36].

##### Duration of TE

The duration of TE was shown to be the key model driver in two analyses from the US [20, 61]. In these analyses with base-case ICERs/QALY of US$47,053 [20] and US$50,195 [61], the model was most sensitive to the duration of TE. If the treatment was assumed only effective for the median duration of the trial (27 months), the ICERs increased to US$120,623 [20] or US$135,815 [61]. Across analyses, the ICERs were less than US$100,000 if the treatment was effective for at least 36 months.

**Fig. 4:**
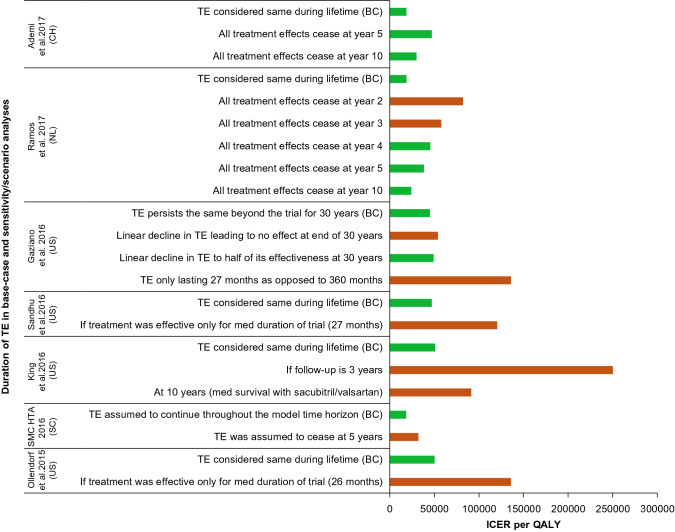
Studies of sacubitril/valsartan evaluating impact of duration of treatment effect on ICERs in base-case and sensitivity/scenario analyses. The green and red bars indicate the ICERs below and above the country-specific WTP thresholds, respectively. The ICERs are presented in the currency of respective country as reported in the publication. *BC* base-case, *CH* Switzerland, *HTA* health technology assessment, *ICER* incremental cost-effectiveness ratio, *NL* the Netherlands, *QALY* quality-adjusted life-year, *SC* Scotland, *SMC* Scottish Medicines Consortium, *TE* treatment effect, *US* United States

The duration of TE was also analyzed using one-way SA in seven other studies (Fig. 4) [18, 25, 33, 35, 43, 59, 60]. When the TE was considered to be restricted to the mean/median duration of the PARADIGM-HF trial, the resulting ICERs were above the country-specific WTP limits. A similar trend was observed even when the TE was assumed to cease at 5 or 10 years, except for one study from Switzerland [25].

##### Time horizon

Time horizon was explored in the SAs of 14 studies (Table 2) [17, 19, 25, 29, 38, 42, 43, 49, 50, 52, 55, 56, 59, 62]. Three of the four model analyses [19, 30, 36, 52] which concluded that sacubitril/valsartan is not a cost-effective therapy modeled shorter time horizons (5 [52], 10 [19], and 15 years [30]) than lifetime in the base-case analysis while other studies with a lifetime horizon typically reported sacubitril/valsartan as cost-effective. A study from the US by Zueger et al. [52] using 5 years’ time horizon in the base-case reported an ICER of US$143,891/QALY. However, scenario analyses using different time horizons showed different ICERs (3 years, US$223,344; 10 years, US$89,824; 30 years, US$67,997) [52]. A study from Singapore with a time horizon of 10 years in the base-case showed sacubitril/valsartan as not cost-effective (ICER SG$74,592) [19], whereas another study with a lifetime horizon concluded sacubitril/valsartan to be cost-effective (ICER SG$37,199) [40] at a WTP level of SG$50,000 per QALY. The time horizon that was adopted impacted the CE conclusions for sacubitril/valsartan in these studies. Given that HF is a chronic condition, the use of a lifetime horizon in CE analysis could be considered to be the most appropriate base-case setting for sacubitril/valsartan economic modeling.

**Table 2:** Studies of sacubitril/valsartan evaluating impact of time horizon on ICERs in base-case and sensitivity/scenario analyses

Study (country)	Base-case		ICER/QALY at time horizons used in sensitivity and scenario analyses							
	Time horizon	ICER/QALY	1 year	2 years	3 years	5 years	10 years	15 years	20 years	30 years
Grant et al. 2020 [55] (CA)	5 years	CA$34,727	–	CA$50,766	–	–	CA$24,426	–	–	–
Wu et al. 2020 [49] (CN)	10 years	$2,481	–	–	–	$3,685	–	$2,033	$1,874	–
Earla and Sansgiry 2019 [30] (US)	15 years	$75,270^#^	–	–	–	–	–	–	–	–
Perera et al. 2019 [56] (AU)	Lifetime	AU$77,889	–	–	–	AU$109,302	AU$78,373	–	–	–
Park et al. 2019 [42] (KR)	Lifetime	$12,722	–	–	–	–	–	$13,751	$12,850	–
Zueger et al. 2018 [52] (US)	5 years	US$142,891	–	–	$223,344	–	$89,824	–	–	$67,997
Zaca 2018 [50] (IT)	10 years	–€98,500/	–	–	–	–	–	–	–	Dominant
Liang et al. 2018 [19] (SG)	10 years	SG$74,592	–	SG$271,291	–	–	–	–	SG$54,777	–
D’Angiolella et al. 2017 [29] (IT)	Lifetime	€19,487	–	–	–	–	€26,180	–	–	–
Ademi et al. 2017 [25] (CH)	Lifetime	CHF25,684	–	CHF58,679	–	–	–	–	–	–
Ramos et al. 2017 [43] (NL)	Lifetime	€17,600	€88,710	–	€55,555	€41,576	€27,350	–	–	–
CADTH (HTA), 2016 [58] (CA)	20 years	CA$29,999	–	–	–	–	CA$42,787	–	–	–
NICE (HTA), 2016 [17] (UK)	Lifetime	£17,939	–	–	–	–	–	–	–	–
SMC (HTA), 2016 [59] (SC)	Lifetime	£18,348	–	–	–	–	–	–	–	–

*CA* Canada, *CH* Switzerland, *CN* China, *HTA* health technology assessment, *ICER* incremental cost-effectiveness ratio, *IT* Italy, *KR* South Korea, *NICE* National Institute for Health and Care Excellence, *NL* the Netherlands, *QALY* quality-adjusted life-year, *SC* Scotland, *SG* Singapore, *SMC* Scottish Medicines Consortium, *US* United States

### CE models of sacubitril/valsartan in inpatient setting

Three models, one each from the US [33], Australia [56], and Russia [57], reported CE analyses of sacubitril/valsartan among in-hospital patients stabilized after an acute decompensation for HF. The US study estimated the CE of inpatient initiation of sacubitril/valsartan versus enalapril compared with no initiation or post-hospitalization initiation of sacubitril/valsartan among stabilized patients with HFrEF. Using data from both the PIONEER-HF and PARADIGM-HF trials, a Markov model was developed with five health states comprising (1) inpatient, (2) ﻿1 month after hospitalization, (3) 2 months after hospitalization, (4) more than 2 months after hospitalization for HF, and (5) death. The base-case analysis included a lifetime horizon from a healthcare and societal perspective. The results indicated that from a healthcare system perspective, initiation of sacubitril/valsartan during hospitalization was associated with per patient annual savings of US$452 (vs continuing enalapril) and US$811 (vs initiation at 2 months after hospitalization). Sacubitril/valsartan was shown to be a cost-effective treatment with an ICER/QALY of US$21,532 compared with continued enalapril treatment over a lifetime, and extendedly dominated a strategy of enalapril initiation during hospitalization followed by later initiation of sacubitril/valsartan. The inpatient initiation of sacubitril/valsartan was reported to be associated with per patient annual savings of US$460 (vs no initiation) and US$813 (vs initiation after hospitalization) when analyzed from a societal perspective. One-way SA showed that the cost of sacubitril/valsartan influenced the results the most, with sacubitril/valsartan being cost saving at an annual price of US$1,043 and ICER/QALY being less than US$100,000 at an annual price of US$3,200. For the ICER to exceed US$100,000 per QALY, the annual cost of sacubitril/valsartan would need to be US$12,760 or more [33].

In the study from Australia, a three-state Markov model (“alive and event-free”, “alive after non-fatal hospitalization for acute decompensated HF” or “dead”) was developed to estimate the clinical progress and costs of patients over a lifetime horizon using data from the PIONEER-HF trial. The model considered the probability of rehospitalization and mortality, and efficacy of sacubitril/valsartan based on percent change in NT-proBNP. Compared with enalapril, treatment with sacubitril/valsartan was shown not to be cost-effective (at a WTP threshold of Australian [AU]$50,000), with an ICER/QALY of AU$77,889. The model was most sensitive to the cost of sacubitril/valsartan as well as the percent change in NT-proBNP in the sacubitril/valsartan arm. The ICERs/QALY obtained by varying the NT-proBNP by ± 15% of the base-case to − 39.7% and − 53.7% were AU$105,634 and AU$59,770, respectively. The authors concluded that more than a 25% reduction in cost (AU$2,584 per year in base–base) would be needed to achieve CE for sacubitril/valsartan initiation in the inpatient setting in Australia [56]. The study from Russia concluded that the use of sacubitril/valsartan in patients with HF hospitalized after an acute decompensation for HF is a cost-effective management strategy that significantly improves the prognosis in this category of patients. More details on model and health states could not be obtained as this study was published in Russian and very limited information was provided in the abstract [57].

### Quality of study

The study quality of all full publications and HTAs was assessed using the Drummond and Jefferson checklist [24]. Abstracts were not appraised owing to the limited methodological information provided in them. Based on the results of the quality assessment, all published studies appeared to be of good quality. Although the quality appraisal of HTAs was performed, the information was limited and a clear inference about their quality could not be drawn (Supplementary Fig. 2).

## Discussion

This SLR provides comprehensive evidence from 44 CE models of sacubitril/valsartan (including three models based on sacubitril/valsartan initiation in inpatient setting) in the treatment of patients with HFrEF published since the launch of sacubitril/valsartan in 2015. Overall, the finding from the majority of economic analyses that were conducted was that sacubitril/valsartan is a cost-effective therapy in patients with chronic HFrEF in the outpatient setting (concluded by ~ 90% of studies). Limited evidence also showed sacubitril/valsartan to be a cost-effective treatment strategy when initiated in the inpatient setting. TE on cardiovascular mortality, all-cause mortality, cost of sacubitril/valsartan, duration of TE and time horizon were shown to be the key CE drivers in the sacubitril/valsartan models.

Several methodological observations can be made based on this SLR. Generally, the included studies employed the widely used Markov models or Markov mixed models (incorporating Markov model and regression models) to predict long-term outcomes. The suitability of using a Markov model structure in modeling chronic diseases such as cardiovascular disease is considered appropriate relative to the use of other structures, e.g., simulation [63]. However, the high diversity adopted in terms of structural assumptions or parameters chosen within the sacubitril/valsartan models limited the comparison of the included studies. Differences in modeling methods were observed with respect to model inputs, duration of TE, description of health states or events, modeling assumptions, time horizons and study perspectives. There is a need to adopt a set of standard parameters in the sacubitril/valsartan models at least with respect to major modeling decision points, which could improve the comparability of results across countries and settings.

This SLR found that the TE of sacubitril/valsartan on cardiovascular mortality, mortality, cost of sacubitril/valsartan, duration of TE and time horizon were model parameters with the highest impact on ICERs. The effect of sacubitril/valsartan on cardiovascular mortality or all-cause mortality was the most frequently identified first-reported model driver in 24 of the 44 CE analyses. The data on TE across model analyses were obtained from the PARADIGM-HF study [10], which can be considered a well-designed trial; thus, it represents a robust, quality source of clinical effect data. Enalapril is the only ACEi to reduce mortality in chronic HFrEF and the mean dose of enalapril achieved in the PARADIGM-HF study was greater than in other trials, yet the benefits of sacubitril/valsartan versus enalapril were statistically and clinically compelling [64]. A recent SLR including data from real-world studies comparing efficacy of sacubitril/valsartan with standard-of-care showed superior efficacy of sacubitril/valsartan in reducing the risk of HF hospitalizations, all-cause hospitalizations, and all-cause mortality in most studies [65]. This suggests that clinical benefits of sacubitril/valsartan in real-world settings are consistent with that observed in PARADIGM-HF trial. The cost of sacubitril/valsartan was a key CE driver in five models, and the duration of TE a driver in two models. Time horizon was evaluated using sensitivity/scenario analysis in several studies, wherein it was evident that the studies with shorter time horizons altered the overall conclusions or provided ICERs above the WTP thresholds of the respective countries.

Notably, the perspective used for economic analysis (e.g., payer, provider, social), sources of costs (e.g., national/private health insurance, hospital-level, personal-level [out-of-pocket]) and utilities also play an important role on the CE results. In this SLR, we did not observe the impact of perspective on CE conclusions of sacubitril/valsartan whereas the evidence is limited for other two parameters and further research is suggested. Most models used the payer’s perspective and three used a societal perspective. All three studies using a societal perspective also concluded sacubitril/valsartan as a cost-effective therapy [20, 26, 43]. None of the studies compared alternative perspectives to evaluate if there are differences in results and no study used perspective as the input parameter in their sensitivity analyses. National/private health insurance cost was the source of costs in most studies. In two studies from Singapore, one used hospital-based costs [19] whereas another used costs from national health insurance [40]; the former study concluded that sacubitril/valsartan is not a cost-effective option, whereas the latter study concluded the opposite. The source of utilities appeared to have an impact on CE results. Utilities were mostly taken from the PARADIGM-HF trial, published literature, or other trials. Of the four studies concluding sacubitril/valsartan may not be a cost-effective treatment, two used utilities from different trials [19, 52] (i.e., SHIFT and CARE-HF). Nevertheless, it is uncertain if the changed conclusions were primarily due to difference in utility sources as these studies used short time horizons as well.

Despite the wide variations adopted in the structural assumptions and parameters selected in the model, sacubitril/valsartan was reported as a cost-effective therapy for the treatment of patients with HFrEF in all studies except four (two US [30, 52], one Singapore [19], and one Thailand [36]). A study from the US by Zueger et al. [52] using enalapril as a comparator and 5 years’ time horizon reported an ICER value of $143,891/QALY gained. The short time horizon used in this study impacted the ICER, which was much different from the ICERs (range: $45,017 [18] to $50,959 [35] per QALY) reported in other studies from the US conducted using the lifetime horizon [18, 20, 33, 35, 61]. Sacubitril/valsartan was not considered likely to be cost-effective at a WTP threshold of $100,000/QALY, although it was at a WTP threshold of $150,000/QALY. Nonetheless, an SA using a time horizon of 30 years resulted in the ICER/QALY of $67,997 [52]. The study by Earla and Sansgiry [30] also used a shorter time horizon (i.e., 15 years) but with a cycle length of 1 year, whereas other studies used a cycle length of 1 or 3 months. This study indicated that sacubitril/valsartan may not be a cost-effective alternative versus enalapril to reduce hospitalizations, with an ICER of $75,279 avoided per hospitalization at a specified WTP of US$50,000 for this outcome [30]. However, this study was published as a conference abstract with limited information on methodology, and the measured outcome (hospitalization) was different from the commonly assessed ICER/QALY measured in other studies. In the study from Singapore by Liang et al. [19], sacubitril/valsartan was not shown to be a cost-effective therapy compared with enalapril at an ICER/QALY of SG$74,592. However, another study from Singapore by Lee et al. [40] demonstrated sacubitril/valsartan as a cost-effective treatment over enalapril with an ICER/QALY of SG$37,199 [40]. The notable differences between the two studies included the time horizon (10 years vs lifetime), price of enalapril, transition probabilities to move patients between NYHA classes taken from the SENIORS study rather than the PARADIGM-HF study, and the utility values collected from the CARE-HF trial instead of the PARADIGM-HF trial [19, 40, 66]. If the study by Liang et al. had used a lifetime horizon, sacubitril/valsartan would have been a cost-effective treatment approach in Singapore. A study from Thailand using a lifetime horizon showed sacubitril/valsartan to be associated with an ICER/QALY of THB162,276, which was slightly above the WTP mark of THB160,000 [36]. The risk of cardiovascular death and cost of sacubitril/valsartan influenced the ICER the most. The authors concluded that sacubitril/valsartan could achieve CE benchmarks with a 2% reduction in its daily price [36].

During the course of our searches, we found three recently published SLRs reporting the CE models for pharmacological interventions in HF [22, 67, 68]. Two of these reviews were published by the same group [22, 67, 68], which provided an update (after June 2010) of the available literature following the publication by Goehler et al. [69] In the first publication, DiTanna et al. [67] synthesized data from 64 publications of different pharmacological therapies (e.g., eplerenone, ramipril, enalapril, ivabradine and sacubitril/valsartan) and showed that a Markov model (used in 44% of publications) was the most commonly used modeling approach in the economic evaluations of interventions in HF. The second publication reporting the key drivers of these models revealed that TEs on mortality or on cardiovascular mortality were the most important parameters [68]. Our findings on the use of the Markov model in the economic analyses of sacubitril/valsartan and the key parameters influencing ICER results are consistent with those reported in the literature. Liu et al. reviewed the economic evaluations of sacubitril/valsartan and included 11 studies published up to August 2019 [22]. Although the review by Liu et al. provides an overview of the model methodology and ICER results, evidence from conference abstracts and HTA submissions and detailed analysis of the model parameters were lacking [22]. Our SLR has the advantage of reporting a data update by incorporating the time span of the past 2 years during which several important papers on CE analyses of sacubitril/valsartan were published.

To the best of our knowledge, this current SLR appears to be more extensive in searches and includes 37 studies that provide 39 CE models and 5 HTA reports of sacubitril/valsartan, with no restriction on publication types per se. Moreover, it comprehensively covers all structural aspects of CE models and parameter choices and their influence on the CE results of sacubitril/valsartan in patients with chronic HFrEF. However, our SLR has some limitations. First, only studies published in English were included. This may be a source of selection bias although the majority of articles are published in English. This SLR also included studies published as conference abstracts; however, they have the limitation of providing inadequate information on the methods or the SA that was conducted. There was great variation in reporting the key information within the included studies; therefore, our analysis was limited to the information provided in the published manuscripts. The authors of published models were not contacted for missing information. However, these limitations are unlikely to have a meaningful effect on the CE results and overall conclusions. Thus, this review highlights some widely used structural assumptions and key CE drivers of the sacubitril/valsartan models. The selection of appropriate input values for key parameters, particularly use of a lifetime horizon in the base-case, should be considered for future economic evaluations.

## Conclusions

The systematic assessment of evidence on the CE models of sacubitril/valsartan showed that sacubitril/valsartan was reported to be a cost-effective therapy in chronic HFrEF patients when initiated in the outpatient setting, with ICERs below the WTP thresholds in 90% of the models. Sacubitril/valsartan was also shown to be a cost-effective treatment strategy when initiated in the inpatient setting, although the evidence is limited and further CE research is warranted. Published CE models differed in structural assumptions and the choice of input parameters. The TE of sacubitril/valsartan on cardiovascular mortality, all-cause mortality, cost of sacubitril/valsartan, duration of TE, and time horizon were the key CE drivers in the economic models of sacubitril/valsartan. Given that HF is a chronic disease, this SLR highlights the need for careful and appropriate selection of model parameters, especially the standard use of a lifetime horizon in the model base-case.

## Supplementary Information

Below is the link to the electronic supplementary material.

Supplementary file1 (DOCX 187 KB)

## Data Availability

The dataset used and analyzed for this study is available upon request.
