# Supplementary material for: Model parameters influencing the cost-effectiveness of sacubitril/valsartan in heart failure: evidence from a systematic literature review
Source: Eur J Health Econ. 2022 Jul 5;24(3):453–67. doi: 10.1007/s10198-022-01485-3 (PMC10060315; doi:10.1007/s10198-022-01485-3)
Supplement: Supplementary file 1 — Supplementary file1 (DOCX 187 KB) [file 10198_2022_1485_MOESM1_ESM.docx]

**Supplementary information**

**Table S1.** **Embase and MEDLINE search strategy using Embase.com interface (searched on July 25, 2021)**

| **Parameter** | **S. No** | **Search Strings** | **Relevant Hits** |
| --- | --- | --- | --- |
| **Disease terms** | **1** | 'heart failure'/syn | 630530 |
|  | **2** | (heart OR cardi* OR myocard*) NEAR/3 (failure* OR insufficien*) | 496756 |
|  | **3** | 'cardiomyopathy'/syn | 177833 |
|  | **4** | cardiomyopath* | 171031 |
|  | **5** | #1 OR #2 OR #3 OR #4 | 742269 |
| **Treatment terms** | **6** | arni OR (neprilysin NEAR/2 (inhibit* OR antagonist*)) | 2677 |
|  | **7** | sacubitril OR sacubitrilat OR lbq657 OR 'lbq 657' OR ahu377 OR 'ahu 377' OR entresto OR lcz696 OR 'lcz 696' | 3378 |
|  | **8** | #6 OR #7 | 4919 |
| **Model and economic analysis terms** | **9** | 'cost minimization analysis'/syn OR ('cost minimi?ation' NEXT/1 analys*) | 4326 |
|  | **10** | 'cost benefit analysis'/syn | 99270 |
|  | **11** | 'cost benefit' NEXT/1 analys* | 90104 |
|  | **12** | 'cost utility' NEXT/1 analys* | 11744 |
|  | **13** | 'cost utility analysis'/syn OR 'economic evaluation'/syn | 324353 |
|  | **14** | 'cost effective*' NEXT/1 analys* | 164396 |
|  | **15** | 'cost effectiveness analysis'/syn | 187203 |
|  | **16** | (economic OR pharmacoeconomic) NEXT/1 (evaluation OR assessment OR analys* OR stud*) | 40228 |
|  | **17** | 'cea' OR 'cma' OR 'cba' OR 'cua' OR 'cca' | 175541 |
|  | **18** | 'decision theory'/syn OR 'decision tree' | 21756 |
|  | **19** | 'decision tree'/syn | 20374 |
|  | **20** | 'economic model' | 4837 |
|  | **21** | markov OR deterministic | 55123 |
|  | **22** | (transition NEXT/1 probabilit*) OR (health NEXT/1 stat*) OR (sensitivity NEXT/1 analys*) OR (health NEXT/1 outcome) | 358907 |
|  | **23** | ('patient level' OR 'discrete event') NEXT/1 simulat* | 1547 |
|  | **24** | 'incremental cost' | 20872 |
|  | **25** | icer OR qaly OR daly OR wtp OR tto | 45183 |
|  | **26** | 'cost offset' | 302 |
|  | **27** | #9 OR #10 OR #11 OR #12 OR #13 OR #14 OR #15 OR #16 OR #17 OR #18 OR #19 OR #20 OR #21 OR #22 OR #23 OR #24 OR #25 OR #26 | 931053 |
| **Combining all three terms**  **(Final hits)** | **28** | #5 AND #8 AND #27 | **239** |

ARNI, angiotensin receptor neprilysin inhibitor; CBA, cost-benefit analysis; CCA, cost-consequence analysis; CEA, cost-effectiveness analysis; CMA, cost-minimization analysis; CUA, cost-utility analysis; DALY, daily adjusted life year; ICER, incremental cost-effectiveness ratio; QALY, quality adjusted life year; TTO, time-trade off; WTP, willingness-to-pay threshold.

**Table S2. MEDLINE Epub ahead of print, In-process and other non-indexed citations search strategy using PubMed.com interface (searched on July 25, 2021)**

| **Parameter** | **S. No** | **Search Strings** | **Relevant Hits** |
| --- | --- | --- | --- |
| **Disease terms** | **1** | heart failure | 275,756 |
|  | **2** | (heart OR cardi* OR myocard*) AND (failure* OR insufficien*) | 439,590 |
|  | **3** | cardiomyopath* | 104,425 |
|  | **4** | #1 OR #2 OR #3 | 511,064 |
| **Treatment terms** | **5** | (arni OR (neprilysin AND (inhibit* OR antagonist*))) | 3,453 |
|  | **6** | (sacubitril OR sacubitrilat OR lbq657 OR 'lbq 657' OR ahu377 OR 'ahu 377' OR entresto OR lcz696 OR 'lcz 696' | 4,733 |
|  | **7** | #5 OR #6 | 7,522 |
| **Model and economic analysis terms** | **8** | (cost minimisation OR cost minimization) | 2,920 |
|  | **9** | cost benefit | 116,054 |
|  | **10** | cost utility | 19,669 |
|  | **11** | cost effectiveness | 138,577 |
|  | **12** | (economic evaluation OR economic evaluations) | 118,481 |
|  | **13** | ((economic OR pharmacoeconomic) AND (evaluation OR assessment OR analys* OR stud*)) | 605,605 |
|  | **14** | (cea OR cma OR cba OR cua OR cca) | 92,358 |
|  | **15** | (decision theory OR decision tree) | 37,501 |
|  | **16** | (economic model OR economic models) | 71,614 |
|  | **17** | (markov OR deterministic) | 46,543 |
|  | **18** | (transition probability OR transition probabilities) | 17,217 |
|  | **19** | (health statistics OR health statistic) | 717,894 |
|  | **20** | (sensitivity analysis OR sensitivity analyses) | 1,066,335 |
|  | **21** | (health outcome OR health outcomes) | 914,085 |
|  | **22** | (patient level simulation OR patient level simulations OR discrete event simulation OR discrete event simulations) | 11,117 |
|  | **23** | (incremental cost OR incremental costs) | 17,664 |
|  | **24** | (icer OR qaly OR daly OR wtp OR tto) | 38,452 |
|  | **25** | cost offset | 4,452 |
|  | **26** | #8 OR #9 OR #10 OR #11 OR #12 OR #13 OR #14 OR #15 OR #16 OR #17 OR #18 OR #19 OR #20 OR #21 OR #22 OR #23 OR #24 OR #25 | 3,074,170 |
| **Combining all three terms** | **27** | #4 AND #7 AND #26 | 746 |
| **Final hits** | **28** | #27 AND (pubstatusaheadofprint OR inprocess[sb]) | **47** |

ARNI, angiotensin receptor neprilysin inhibitor; CBA, cost-benefit analysis; CCA, cost-consequence analysis; CEA, cost-effectiveness analysis; CMA, cost-minimization analysis; CUA, cost-utility analysis; DALY, daily adjusted life year; ICER, incremental cost-effectiveness ratio; QALY, quality adjusted life year; TTO, time-trade off; WTP, willingness-to-pay threshold.

**Table S3. Inclusion and exclusion criteria for this systematic literature review**

| **Inclusion criteria** | **Exclusion criteria** |
| --- | --- |
| **Patient Population:**   - Patients (≥18 years) having chronic heart failure | - Any other patient population |
| **Intervention:**   - Sacubitril/valsartan | - Any other interventions |
| **Comparators:**   - Placebo - Any active comparator | - None |
| **Outcomes:**   - Study details: - Study objective/hypothesis - Country - Type of economic analysis - Perspective (healthcare/societal/payer) - Type of model (cohort, individual patient simulation) - Model details - Health states or events (first/repeat events) - Time horizon - Cycle length - Discounting - Model inputs - Population - Description of model or cost assumptions including, but not limited to: - Clinical input parameters and sources (e.g., HF hospitalizations, myocardial infarction, stroke) - Cost and resource use input parameters and sources (e.g., hospitalizations) - QoL input parameters and sources (e.g., baseline utility levels, or utility decrements for adverse events) - Treatment discontinuation - Health outcomes: - Quality-adjusted life years gained (QALYG) - Life years gained (LYG) - Hospitalizations predicted in treatment arm - Hospitalizations avoided - Cost-effectiveness ratios: - Incremental cost-effectiveness ratios (ICERs) per LYG or per QALYG | - Studies not reporting relevant outcomes of interest |
| **Study designs:**   - Economic evaluations - Cost-benefit analysis - Cost-effectiveness analysis - Cost-utility analysis - Cost-minimization analysis - Cost-consequence analysis | - Budget impact model - Reviews, systematic review, letters, editorials and single-case studies |
| **Language:**   - English (English abstracts of non-English publications were included) | - Non-English |
| **Country:**   - No country restriction | - None |
| **Publication date:**   - Journal articles: Published up to July 25, 21 - Conference abstracts: Published up to July 25, 2021 | - None |

**Table S4. Overview of included publications and HTA reports**

| **Study, Country** | **HF type,**  **Population,**  **Age** | **Comparator** | **Model,**  **Economic analysis type** | **Health states** | **Time horizon,**  **Cycle length** | **Perspective,**  **Discount** | **Source of Cost data** | **Source of Utility data** | **Results**  **(base case ICER)** | **Sensitivity analysis** | **Parameters considered in one-way SA** | **Scenario analysis** |
| --- | --- | --- | --- | --- | --- | --- | --- | --- | --- | --- | --- | --- |
| Gokhale et al. 2021^54^ (India) | Patients with HFrEF,  Not reported,  Not reported | Enalapril | Unclear,  Cost-consequence analysis | Mortality risk reduction; HF hospitalization; length of stay | 6, 12, 24 and 27 months,  Not reported | Indian healthcare setting,  Not reported | India Wage Report, 2018  Published literature | Not Reported | Annual cost saving of INR43,290 | Not reported | Not applicable | Not reported |
| Gaziano et al. 2020^33^ (US) | Patients hospitalized with HFrEF (ADHF),  Not reported,  63.8 years | Enalapril | Markov model (5-states),  CUA | Inpatient; 1 month after hospitalization; 2 months after hospitalization; >2 months after hospitalization for HF; death | Lifetime,  Not reported | Healthcare payer, Societal (both),  3%  (both costs and health outcomes) | Red Book, 2019  DRG Expert 2014  Published literature | PARADIGM-HF | USD 21,532/ QALY (Payer),  Cost saving (Societal) | One-way,  Two-way,  PSA | Cost of sac/val; Cost of HF admission; Cost of comparator; TE on HF admission; Incremental utility; Cost of in-hospital sac/val initiation | TE tapered after 29 months or 10 years |
| Grant et al. 2020^55^ (Canada) | Patient with HFrEF,  Not reported,  64 years | Enalapril | Markov model (3-states),  CUA | Alive on ARNI; alive on ACEi; death | 5 years,  1 month | Canadian public health payer,  1.5%  (both costs and health outcomes) | Alberta Drug Benefit List  Canadian Institute for Health Information. Patient Cost Estimator  Schedule of Medical Benefits | PARADIGM HF | CA$34,727/QALY (*De novo* initiation),  CA$35,871/QALY (Early initiation),  CA$40,234/QALY (Late initiation) | One-way, PSA | Cost of sac/val; HR of death; Cost of HF hospitalization; HR of HF hospitalization; Utility-alive on sac/val; Utility-alive on enalapril; monthly HF hospitalization rate; monthly death rate; monthly cost of enalapril; cost of transthoracic echocardiogram; disutility of HF hospitalization; discount rate | Guideline-based ARNI initiation; time horizons (27 months; 10 years) |
| Borges et al. 2020^26^ (Portugal) | HFrEF,  NYHA class II-IV,  Not reported | Enalapril | Markov model [2-state] (regression-based models),  CUA | Alive (hospitalization, HRQoL); death | 30 years,  1 month (with half cycle correction) | Societal,  5%  (both costs and health outcomes) | INFARMED. Infomed, 2016  Published literature | Published literature | €22,702/QALY | One-way, PSA | CV mortality (coefficient of constant); CV mortality (coefficient of age^2); CV mortality (coefficient of sac/val); Utility (coefficient of sac/val); CV mortality (coefficient of beta blocker use); Hospitalization (coefficient of constant); Hospitalization (coefficient of sac/val); Hospitalization (coefficient of age^2); CV mortality (coefficient of gamma); CV mortality (coefficient of Region [APAC vs North America]) | No additional benefit of sac/val in utilities; Western Europe patients; All-cause mortality using data from PARADIGM-HF; Increase in non-CV mortality: SMR = 2 (instead of 1); Sac/val and enalapril costs according to  target doses; 50% reduction in hospitalization; Baseline NYHA class III/IV; Prior use of ACE inhibitor; Baseline NYHA class I/II; Prior use of ARB; Discount rate: 0% |
| Chin et al. 2020^27^ (Australia) | HFrEF,  Not reported,  63 years | Enalapril | Markov model (2-state),  CEA and CUA | Alive (no HF hospitalization); non-fatal HF; CV death; non-CV death | 20 years,  1 year | Australian healthcare system,  5%  (both costs and health outcomes) | Independent Hospital Pricing Authority (IHPA), 2014 | Published literature | AUD 40,513/  QALY | One-way, PSA | CV death (comparator); TE on CV death; Non-CV death (comparator); TE on non-CV death; Utility input; cost of non-CV death; Non-fatal HF hospitalization (comparator); TE on non-fatal hospitalization; cost of CV death; cost of background therapy | Not reported |
| Wu et al. 2020^49^ (China) | HFrEF,  Not reported,  64 years | Enalapril | Markov model,  CUA | NYHA class I-IV; death | 10 years,  1 month | Chinese patients’ perspective,  3.5%  (both costs and health outcomes) | Published literature | Published literature | $2,481/QALY | One-way, Two-way,  PSA | Cost of sac/val; Cost of comparator; TE on CV death; Baseline probability for CV death (comparator); Cost of doctor visit; Utility of NYHA class II; Probability of NYHA progress (comparator); Baseline probability of hospitalization (comparator); Probability of NYHA progression (sac/val); discount rate; Co-payment; Utility of NYHA class III; Risk of CV death (NYHA III vs II); Cost of hospitalization; Cost of readmission; Utility of NYHA I; Utility of NYHA IV; Disutility | Time horizon (5, 15 and 20 years) |
| Zyryanov et al. 2020^57^ (Russia) | Patients with ADHF,  Not reported,  Not reported | Enalapril | Markov model,  CEA | Unclear | Unclear | Russian Federation health care system,  Unclear | Not reported | Not reported | RUB307,294 per LYG | Unclear | Unclear | Unclear |
| *Earla and Sansgiry 2019^30^ (US) | HFrEF.  Not reported,  Not reported | Enalapril | Markov model (5-states),  CEA | Not reported | 15 years,  1 year | Third-party payer,  3%  (costs only) | Not reported | Not reported | USD 75,279 to avoid one hospitalization | PSA | Not reported | Not reported |
| van der Pol et al. 2019^48^ (Germany) | HFrEF,  Not reported,  64 years | Enalapril,  Placebo | Markov model,  CUA | OP treated HFrEF; hospitalization to general ward; hospitalization to ICU; death | 30 years,  1 month | Healthcare payer,  3%  (both costs and health outcomes) | German costs data | PARADIGM-HF | €19,300/QALY (vs enalapril),  €9,900/QALY (vs placebo) | One-way, PSA | TE on mortality; Utility of non-hospitalization; TE on hospitalization; TE on ICU hospitalization; Cost of hospitalization; Cost of comparator (enalapril); Utility in hospital; Cost of OP treatment | Age 55 and 75 years; Discount 0% and 5% |
| Perera et al. 2019^56^ (Australia) | Patients with ADHF,  Not reported,  61 years | Enalapril | Markov model (3-states),  CEA and CUA | Alive and event-free (no  re-hospitalisation for ADHF); alive after non-fatal hospitalisation  for ADHF; death | Lifetime (15 years),  First cycle 8 weeks and subsequent cycles 1 year | Australian healthcare perspective,  5%  (both costs and health outcomes) | Pharmaceutical Benefits Scheme (PBS)  Published literature | Published literature | AU$77,889/QALY | One-way, PSA |  | Discount (0% and 3.5%); time horizons (5 and 10 years); age-related trends; cost of sac/val (10%, 25% and 50% reduction); baseline NT-proBNP; NT-proBNP at one year |
| Park et al. 2019^42^ (South Korea) | HFrEF,  LVEF <35% and NYHA class II-III  60 years | Enalapril,  ARBs | Markov model (regression-based models),  CEA and CUA | Alive (hospitalization, AE); death | Lifetime (30 years),  1 month | Korean healthcare perspective,  5%  (both costs and health outcomes) | Published literature | Published literature | USD12,722/QALY (vs Enalapril),  USD11,970/QALY (vs ARBs) | One-way, PSA | Daily cost of sac/val; Discount rate; CV mortality (with Weibull and Log-logistic); Cost of hospitalization; Time horizon (15 and 20 years); Utility weights; Comparator (SoC) | Not reported |
| Zueger et al. 2018^52^ (US) | HFrEF,  NYHA class II-IV,  Not reported | Enalapril | Markov model,  CUA | NYHA class II-IV; death | 5 years,  1 month | Payer,  3%  (both costs and health outcomes) | Medi-Span Price Rx, 2017  Red Book, 2017  Published literature | SHIFT trial  Published literature | USD 143,891/ QALY | One-way, Two-way, PSA | TE on mortality; Probability of NYHA progression (comparator); Cost of sac/val; Probability of NYHA progression (sac/val); Probability of mortality (comparator); Treatment discontinuation due to non-tolerability (comparator); Discount rate; Cost of comparator; TE on mortality (comparator vs. alternate therapy); Cost of chronic care for NYHA II HF; Treatment discontinuation due to non-tolerability (sac/val); Utility in NYHA II; Utility in NYHA IV; Cost of chronic care for NYHA IV; Cost of HF hospitalization; TE on progression of NYHA stage; Cost of chronic care for NYHA III HF; Utility in NYHA III | Time horizon (3, 10 and 30 years); NYHA distribution (90% in II and 10% in III; 25% in II, 50% in III and 25% in IV) |
| Lacey et al. 2018^39^ (Brazil) | HFrEF,  Not reported,  Not reported, | Enalapril | Markov model (regression-based models),  CUA | Alive (hospitalization rates, HRQoL, AE rates); death | Lifetime (30 years),  1 month | Brazilian Health system,  5%  (both costs and health outcomes) | Drug Market Regulation Chamber, 2017  Brazil's Health Price Database, 2016-2017  PARADIGM-HF  SIGTAP, 2017  DATASUS, 2016-2017  Published literature | PARADIGM-HF | BRL 28,154/ QALY | One-way,  PSA | CV mortality (coefficient of constant); CV mortality (coefficient of sac/val); CV mortality (coefficient of age^2); Discount rate on health outcomes; Discount rate on costs; CV mortality (coefficient for region [Latin Am vs North Am); Utility (coefficient of sac/val); CV mortality (coefficient of beta blocker use); CV mortality (coefficient for Race [Other vs. Caucasian]); Hospitalization (coefficient of constant); | Not reported |
| McMurray et al. 2018^41^ (Colombia) | HFrEF,  Not reported,  Not reported, | Enalapril | Markov model (regression-based models),  CUA | Alive (hospitalization rates, HRQoL, AE rates); death | Lifetime,  1 month | Healthcare payer,  5%  (both costs and health outcomes) | SISMED 2016 | PARADIGM-HF  Published literature | COP 39,522,754/ QALY (€11,200) | One-way,  PSA | CV mortality (coefficient of constant); CV mortality (coefficient of age^2); CV mortality (coefficient of sac/val); Utility (coefficient of sac/val); Hospitalization (coefficient of constant); Hospitalization (coefficient of age^2); Hospitalization (coefficient of sac/val); CV mortality (coefficient of beta blocker use); Cost of comparator; CV mortality (coefficient of gamma) | Not reported |
| Lacey et al. 2018^38^ (Costa-Rica) | HFrEF,  Not reported,  Not reported, | Enalapril | Markov model (regression-based models),  CUA | Alive (hospitalization rates, HRQoL, AE rates); death | Lifetime (30 years),  1 month | Costa-Rican healthcare,  5%  (both costs and health outcomes) | Costa-Rica specific economic data | PARADIGM-HF | ₡6,108,752/  QALY | One-way,  PSA | CV mortality (coefficient of constant); CV mortality (coefficient of sac/val); CV mortality (coefficient of age^2); Hospitalization (coefficient of sac/val); Hospitalization (coefficient of constant); Hospitalization (coefficient of age^2); Utility (coefficient of sac/val); CV mortality (coefficient for region [Latin Am vs North Am); CV mortality (coefficient of beta blocker use); GP visit (cost/contact) | Not reported |
| McMurray et al. 2018^41^ (UK) | HFrEF,  Not reported,  Not reported, | Enalapril | Markov model (regression-based models),  CUA | Alive (hospitalization rates, HRQoL, AE rates); death | Lifetime,  1 month | Healthcare payer,  3.5%  (both costs and health outcomes) | NHS National Schedule of Reference Costs, 2013-2014 | PARADIGM-HF  Published literature | ₤17,134/QALY (€20,400) | One-way,  PSA | All-cause mortality (Gompertz, coefficient of sac/val); All-cause mortality (Gompertz, coefficient of constant); All-cause mortality (Gompertz, coefficient of age^2); Utility (coefficient of sac/val); Hospitalization (coefficient of constant); Hospitalization (coefficient of age^2); Hospitalization (coefficient of sac/val); All-cause mortality (Gompertz, coefficient of beta blocker use); All-cause mortality (Gompertz, coefficient of gamma); All-cause mortality (coefficient of region) | Not reported |
| Gandjour and Ostwald 2018^32^ (Germany) | HFrEF,  LVEF ≤40% and NYHA class II-IV  64 years | ACE inhibitor | Markov model (2-state),  CEA and CUA | Alive (hospitalization, ED visit); death | 36 years,  1 year | Healthcare payer,  3%  (both costs and health outcomes) | Statistisches Bundesamt, 2015  Fallpauschalen-Katalog 2015  Published literature | PARADIGM-HF | €26,278/QALY | One-way,  Two-way, PSA | TE on mortality; Diminishing TE on mortality; Utility of HF; Baseline risk of hospitalization (comparator); Discount rate; Utility decrement after 3 years for comparator; TE on hospitalization; Baseline risk of mortality (comparator); Utility increment after 3 years for sac/val; Price discount after generic entry (14; 11; 12); Cost of ED visit; TE on ED visit; Baseline risk of ED visit for HF (comparator) | Age 72 years |
| Zaca 2018^50^ (Italy) | HFrEF,  LVEF ≤35% and NYHA class II-III  63.8 years | ICD | Markov model,  CEA and CUA | Stable HFrEF; HF hospitalization; ICD-related complications (for ICD only); death | 10 years,  1 year | Healthcare payer,  3%  (both costs and health outcomes) | Gazzetta Ufficiale, 2013  Published literature | PARADIGM-HF  Published literature | −€98,500/QALY  (dominant) | One-way, PSA | TE on mortality; ICD longevity; Cost of ICD; Cost of sac/val; Utility increment from sac/val; TE on HF hospitalization; Cost of HF hospitalization | Time horizon 30 years |
| McMurray et al. 2018^41^ (Denmark) | HFrEF,  Not reported,  Not reported, | Enalapril | Markov model (regression-based models),  CUA | Alive (hospitalization rates, HRQoL, AE rates); death | Lifetime,  1 month | Healthcare payer,  3%  (both costs and health outcomes) | DRG-takster 2015 | PARADIGM-HF  Published literature | Kr 173,994/  QALY (€22,620) | One-way,  PSA | CV mortality (coefficient of age^2); CV mortality (coefficient of constant); CV mortality (coefficient of sac/val); Hospitalization (coefficient of age^2); Hospitalization (coefficient of constant); Hospitalization (coefficient of sac/val); Utility (coefficient of sac/val); CV mortality (coefficient of beta blocker use); CV mortality (coefficient of age); CV mortality (coefficient of gamma) | Not reported |
| Liang et al. 2018^19^ (Singapore) | HFrEF,  NYHA class II-IV,  66 years | Enalapril | Markov model,  CUA | NYHA class I-IV; death | 10 years,  1 month | Singapore healthcare,  3%  (both costs and health outcomes) | Local hospital | CARE-HF | SGD 74,592/  QALY | One-way, PSA | TE on CV death; Time horizon; Cost of sac/val; Standardized mortality ratio; TE on HF hospitalization; Utility of NYHA II; Utility of NYHA III; Cost of HF hospitalization; Cost of comparator; Utility of NYHA I; Cost of readmission; Cost of inpatient death; Utility for HF hospitalization; Utility of readmission | Not reported |
| *Lee et al. 2018^40^ (Singapore) | HFrEF,  Not reported,  Not reported | Enalapril | Markov model (regression-based models),  CEA and CUA | Hospitalizations; AEs; death | Lifetime (30 years),  1 month | Not reported,  Not reported, | Singapore specific economic data | PARADIGM-HF | SGD37,199/QALY | One-Way | CV mortality; TE of sac/val; impact of age on risk of CV mortality; baseline CV mortality risk | Not reported |
| Krittyaphong et al. 2018^36^ (Thailand) | HFrEF,  LVEF ≤40% and NYHA class II-III  60 years | Enalapril | Markov model,  CEA and CUA | Alive with HFrEF; hospitalization; CV death; Non-CV death | Lifetime,  3 months | Thailand healthcare perspective,  3%  (both costs and health outcomes) | Enalapril: Drug and Medical Supply Information Center, Ministry of Public Health. 2017  Siriraj hospital database | PARADIGM-HF  Published literature | THB 162,276/ QALY | One-way, PSA | Baseline risk of CV non-hospitalization death for comparator; Baseline risk of CV non-hospitalization death for sac/val; Cost of sac/val; Cost of HF hospitalization; Discount rate; Utility of HF for sac/val; Utility of HF for comparator; Utility of hospitalization for comparator; Cost of comparator; Utility of hospitalization for sac/val; Baseline risk of HF hospitalization for comparator; Baseline risk of HF hospitalization for sac/val; Baseline risk of CV hospitalization death for comparator; Baseline risk of CV hospitalization death for sac/val; Risk of readmission for comparator; RR for readmission |  |
| *Zyryanov et al. 2018^53^ (Russia) | CHF patients, with T2DM and ACEi intolerance,  Not reported | Routine care | Markov model,  CUA | Not reported | 3 years,  Not reported | Russian healthcare perspective,  Not reported | Not reported | Not reported | Sac/val: RUB301,146 cost per QALY  Routine care: RUB510,621 cost per QALY | One-way | Not reported | Not reported |
| D’Angiolella et al. 2017^29^ (Italy) | HFrEF,  Not reported,  71 years | Enalapril | Markov model (2-state),  CUA | Alive (HF without complication, hospitalization); death | Lifetime,  1 month | Healthcare (NHS) payer,  3.5%  (both costs and health outcomes) | Italian Medicines Agency, 2016  Gazzetta Ufficiale della Repubblica Italiana, 2017  Published literature | PARADIGM-HF | €19,487/QALY | One-way,  PSA | TE on CV mortality; TE on hospitalization; Baseline risk of hospitalization (comparator) | NYHA class I or II and III or IV; Time horizon 10 years |
| Ademi et al. 2017^25^ (Switzerland) | HFrEF,  LVEF≦ 40%, NYHA  class: II~IV, BNP≧150 pg/mL, or  hospitalization for HF within the last 12 months and a BNP of ≥  100 pg/mL  64 years | Enalapril | Markov model (regression-based models),  CEA and CUA | Alive (hospitalization and AEs, HRQoL); death | Lifetime,  1 month | Healthcare payer,  3%  (both costs and health outcomes) | Swiss Federal Office of Public Health (SFOPH) data relevant to 2015  PARADIGM-HF trial to relevant Swiss DRG codes | PARADIGM-HF | CHF 25,684/ QALY | One-way,  PSA | All-cause mortality for sac/val; Hospitalization (coefficient of constant); Hospitalization (coefficient of age^2); Hospitalization (coefficient of sac/val); All-cause mortality (coefficient of constant); All-cause mortality (coefficient of age^2); Utility (coefficient of sac/val); Hospitalization (coefficient of beta-blocker); Cost of treatment | Discount rate: 1.5% benefits, 6% costs; Time horizon 2 years; HRQoL time trend; TE cease at year 5, 10; No benefit in HRQoL for sac/val |
| van der Pol et al. 2017^21^ (The Netherlands) | HFrEF,  Not reported,  65 years | Enalapril | Markov model,  CEA and CUA | HF (home care only); ward hospitalization; ICU hospitalization; death | Lifetime,  1 month | Healthcare payer,  4% (costs) and 1.5% (health outcomes) | Zorginstituut Nederland. Medicijnkosten  College Van Zorgverzekeringen, 2010 | SHIFT trial | €19,113/QALY | One-way,  PSA | TE on mortality; Utility of HF; Cost of sac/val; Hospital LOS for comparator; Hospital LOS for sac/val; TE on hospitalization; TE on ICU; Elderly care costs; Utility for hospitalization; Cost of hospitalization (per day); Hospital LOS for patients with ICU admission; ICU LOS for patients with ICU admission; Cost of comparator; First-line costs; Costs of other medicine | Not reported |
| Ramos et al. 2017^43^ (The Netherlands) | HFrEF,  Not reported,  75 years | Enalapril | Markov model (regression-based models),  CEA and CUA | Alive (hospitalization rates, HRQoL, AE rates); death | Lifetime,  1 month | Societal,  4% (costs) and 1.5% (health outcomes) | Medicine cost: Z-index  Published literature | PARADIGM-HF | €17,600/QALY | One-way,  PSA | CV mortality (coefficient of age^2); CV mortality (coefficient of constant); CV mortality (coefficient of sac/val); CV mortality (coefficient of age); Hospitalization (coefficient of age^2); Hospitalization (coefficient of sac/val); Hospitalization (coefficient of constant); CV mortality (coefficient of LVEF); CV mortality (coefficient of region); Utility (coefficient of sac/val) | Various subgroups and scenarios were analyzed |
| *Sarioz et al. 2017^45^ (Turkey) | HFrEF,  Not reported,  62.3 years | ACE inhibitor,  ARB | Markov model (regression-based models),  CEA | Not reported | 30 years,  Not reported | Payer’s,  Not reported | Not reported | Not reported | €7,086/LY (vs ACE inhibitor),  €7,021/LY (vs ARB) | Not reported | Not reported | Not reported |
| *Fann et al. 2017^31^ (Taiwan) | HFrEF,  Not reported,  Not reported | Enalapril,  Ramipril,  Perindopril,  Lisinopril | Markov model (2-state),  CEA and CUA | Alive; death | Lifetime,  Not reported | Payer’s perspective,  3%  (both costs and health outcomes) | National Health Insurance Administration (NHIA) claimed data | Not reported | USD18,499/LYG  (vs Enalapril),  USD17,750/LYG (vs Ramipril),  USD17,638/LYG (vs Perindopril),  USD17,774/LYG (vs Lisinopril) | One-way | CV mortality (coefficient of constant) | Not reported |
| Gaziano et al. 2016^18^ (US) | HFrEF,  LVEF ≤40% and NYHA class II-IV,  63.8 years | Enalapril | Markov model (2-state),  CUA | HF (without complication, hospitalization); death | 30 years,  Not reported | Not reported,  3%  (both costs and health outcomes) | DRG 2014  Agency for Healthcare Research and Quality. 2012, Pfuntner 2011  Red Book Montvale. NJ Thomson PDR; 2014  Published literature | PARADIGM-HF | USD 45,017/ QALY | One-way, PSA | TE on mortality; Cost of sac/val; Cost of comparator; TE on HF hospitalization; Incremental utility; Cost of HF hospitalization | Linear decay in TE to no benefit at 30 years; Linear decay in TE to half benefit at 30 years; TE lasting 27 months as opposed to 360 months |
| King et al. 2016^35^ (US) | HFrEF,  Not reported,  60 years | Enalapril | Markov model,  CEA and CUA | NYHA class I-IV; death | Lifetime (40 years),  3 months | Third-party payer,  3%  (both costs and health outcomes) | Red Book Online, 2015  Published literature | CARE-HF trial  Published literature | USD 50,959/ QALY | One-way, Two-way, PSA | CV mortality risk of sac/val; CV mortality risk of comparator; Cost of sac/val; HF hospitalization risk of sac/val; HF risk of comparator; Cost of HF hospitalization; Utility of NYHA II; Cost of 30-day readmission; Utility of NYHA III; Cost of comparator | Duration of treatment: 3, 9 and 10 years |
| Sandhu et al. 2016^20^ (US) | HFrEF,  LVEF ≤40% and NYHA class II-IV,  64 years | Lisinopril | Markov model,  CEA and CUA | Hospitalization (HF, non-HF); ED visit for HF; treatment intolerance; death (CV, non-CV) | Lifetime,  1 month | Societal,  3%  (both costs and health outcomes) | Red Book Online, 2015  Published literature | PARADIGM-HF  Published literature | USD 47,053/ QALY  (NYHA class II: USD 44,351; III/IV: USD 58,194/  QALY) | One-way, Two-way, PSA | Duration of TE; Cost of sac/val; TE of CV death; Utility of HF; Baseline risk of CV death; Baseline risk of non-CV death; Cost of CV death; Incremental utility; TE of HF hospitalization; Cost of HF hospitalization | Subgroup analyses for NYHA class II and III/IV; age (54 and 74 years); treatment tolerance (hospitalization for angioedema) |
| Zaour et al. 2016^51^ (Canada) | HFrEF,  NYHA class II-III,  Not reported | ACE inhibitor | Markov model,  CUA | NYHA class I-IV; death | 20 years,  4 months (for first 3 years) | Canadian healthcare system,  Not reported | Clinical trials, published literature, expert opinion, and standard Canadian sources | PARADIGM-HF | CAD 29,999/ QALY | Not reported | Not reported | Not reported |
| CADTH HTA, 2016^58^ (Canada) | HFrEF,  NYHA class II-III,  75 years | Enalapril | Markov model,  CUA | NYHA class I-IV; death | 20 years,  4 months (for first 3 years) | Canadian healthcare system,  Not reported | Not reported | PARADIGM-HF | CAD 29,999/ QALY | Not reported | Not reported | Not reported |
| *Rojas et al. 2016^44^ (Chile) | HFrEF,  Not reported,  Not reported, | Enalapril | Markov model,  CUA | Not reported | 30 years,  Not reported | Chilean public healthcare,  3%  (both costs and health outcomes) | Not reported | PARADIGM-HF | USD 33,244/  QALY  (USD 29,532/ QALY in Latin Am patients) | PSA | Not reported | Not reported |
| NICE HTA, 2016^17^ (UK) | HFrEF,  LVEF ≤35%, NYHA class II-IV, and who were already taking a stable dose of ACE inhibitor or ARB  64 years | Enalapril,  ARB (candesartan) | Markov model,  CUA | All-cause mortality, all-cause hospitalization; HRQoL; AEs | Lifetime,  1 month (with a half-cycle correction) | NHS and personal social services,  3.5%  (both costs and health outcomes) | NHS National Schedule of Reference Costs, 2013-2014  PSSRU 2014 | PARADIGM-HF | £17,939/QALY (vs ACE inhibitor)  £16,481/QALY (vs ARB) | One-way, PSA | TE on all-cause mortality; Baseline risk of all-cause mortality; Age; Incremental utility; TE on hospitalization | CV mortality (instead of all-cause mortality); Age 75 years; Ramipril as comparator; Time horizon 5 years; TE persisted for less than 5 years |
| *Lacasa et al. 2016^37^ (Spain) | HFrEF,  NYHA class II-IV,  Not reported | Enalapril | Markov model (2-state),  CUA | Alive (on treatment); death | Lifetime,  1 month | Healthcare (NHS) payer,  3%  (both costs and health outcomes) | Spanish published sources | PARADIGM-HF | <€25,000/QALY | PSA | Not reported | Not reported |
| SMC HTA, 2016^59^ (Scotland) | HFrEF,  Not reported,  Not reported | Enalapril,  ARBs | Markov model [2-state] (regression-based models),  CUA | Alive (hospitalization, HRQoL, AEs); death | Lifetime (30 years),  Not reported | Scottish NHA payer,  Not reported | PARADIGM-HF | PARADIGM-HF | £18,348/QALY (vs enalapril),  £16,621/QALY (vs ARBs) | One-way | TE on mortality; Duration of TE; Time horizon; No additional benefit in incremental utility for sac/val | Mortality benefit reduced by 10%, 20% and 30%; Time horizon 5 years; Non-CV mortality using Scottish life tables |
| *Stafylas et al. 2016^47^ (Greece) | HFrEF,  Not reported,  Not reported | Enalapril | Markov model (regression-based models),  CEA and CUA | Alive; death | Lifetime,  Not reported | Greek healthcare payer,  3.5% (both costs and health outcomes) | Greek health social security system, based on ex-factory price list | Not reported | €15,992/QALY | One-way,  PSA | Not reported | Not reported |
| *Costa-Scharplatz et al. 2016^28^ (Sweden) | HFrEF,  Not reported,  Not reported, | Enalapril | Markov model (regression-based models),  CUA | Hospitalization; AEs; death | Lifetime,  Not reported | Swedish healthcare,  3%  (both costs and health outcomes) | Published Swedish sources | PARADIGM-HF | SEK 224,885/ QALY  (€ 24,195/ QALY) | One-way,  PSA | Not reported | Not reported |
| *Spinar et al. 2016^46^ (Czech Republic) | HFrEF,  Not reported,  Not reported | Enalapril | Markov model (regression-based models),  CUA | Alive; death | Lifetime,  1 month | Czech healthcare system,  3%  (both costs and health outcomes) | Local lists of reimbursed medicines, procedures and DRG codes | PARADIGM-HF | €24,300/QALY (PARADIGM-HF)  €26,200/QALY (ESC-HF registry, Czech sub-group)  €29,100/QALY (AHEAD registry of cute HF) | PSA | Not reported | Not reported |
| *Gundersen et al. 2016^34^ (Norway) | HFrEF,  Not reported,  Not reported | Enalapril | Markov model,  CEA and CUA | Not reported | Lifetime,  Not reported | Not reported,  4%  (both costs and health outcomes) | Not reported | Not reported | €23,277/QALY | One-way | Not reported | Not reported |
| PBAC HTA, 2016^60^ (Australia) | HFrEF,  NYHA class II-IV,  75 years | Enalapril | Markov model,  CUA | Alive with HF; death | 10 years,  1 month | Australian healthcare system,  Not reported | Not reported | Not reported | AUD15,000-45,000/QALY | One-way | Risk of CV death; Time horizon; Duration of TE | Not reported |
| #ICER HTA, 2015^61^ (US) | HFrEF,  LVEF ≤40% and NYHA class II-IV,  64 years | Lisinopril | Markov model,  CUA | Hospitalization (HF, non-HF, for angioedema); ED visit for HF; treatment intolerance; death (CV, non-CV) | Lifetime,  1 month | Third-party payer,  3%  (both costs and health outcomes) | Agency for Healthcare Research and Quality national inpatient sample | PARADIGM-HF | USD 50,195/ QALY  (NYHA class II: USD 48,802; III/IV: USD 64,957/  QALY) | One-way, PSA | Duration of TE; Cost of sac/val; TE of CV death; Utility of HF; Incremental utility; Baseline risk of CV death; TE on non-CV death; Cost of CV death; TE of HF hospitalization; Cost of HF hospitalization | Subgroup analyses for NYHA class II and III/IV |

*Indicates the studies published in the form of abstracts.

**Abbreviations:** ACEi, angiotensin-converting enzyme inhibitor; CADTH, Canadian Agency for Drugs and Technologies in Health; CEA, cost-effectiveness analysis; CHF, chronic heart failure; CUA, cost-utility analysis; CV, cardiovascular; ED, emergency department; HF, heart failure; HFrEF, heart failure with reduced ejection fraction; HR, hazard ratio; HRQoL, health-related quality of life; HTA, health technology assessment; ICER, incremental cost-effectiveness ratio; ICD, implantable cardioverter-defibrillator; LVEF, left ventricular ejection fraction; NICE, National Institute for Health and Care Excellence; NYHA, New York Heart Association; PBAC, Pharmaceutical Benefits Advisory Committee; PSA, probabilistic sensitivity analysis; QALY, quality-adjusted life-year; SA, sensitivity analysis; sac/val, sacubitril/valsartan; RR, relative risk; SMC, Scottish Medicines Consortium; T2DM, type 2 diabetes mellitus; TE, treatment effect; UK, United Kingdom; US, United States.

**Figure 1. Model parameters considered in sensitivity/scenario analyses in cost-effectiveness models of sacubitril/valsartan**

**(A) Markov model with constant transition probabilities**


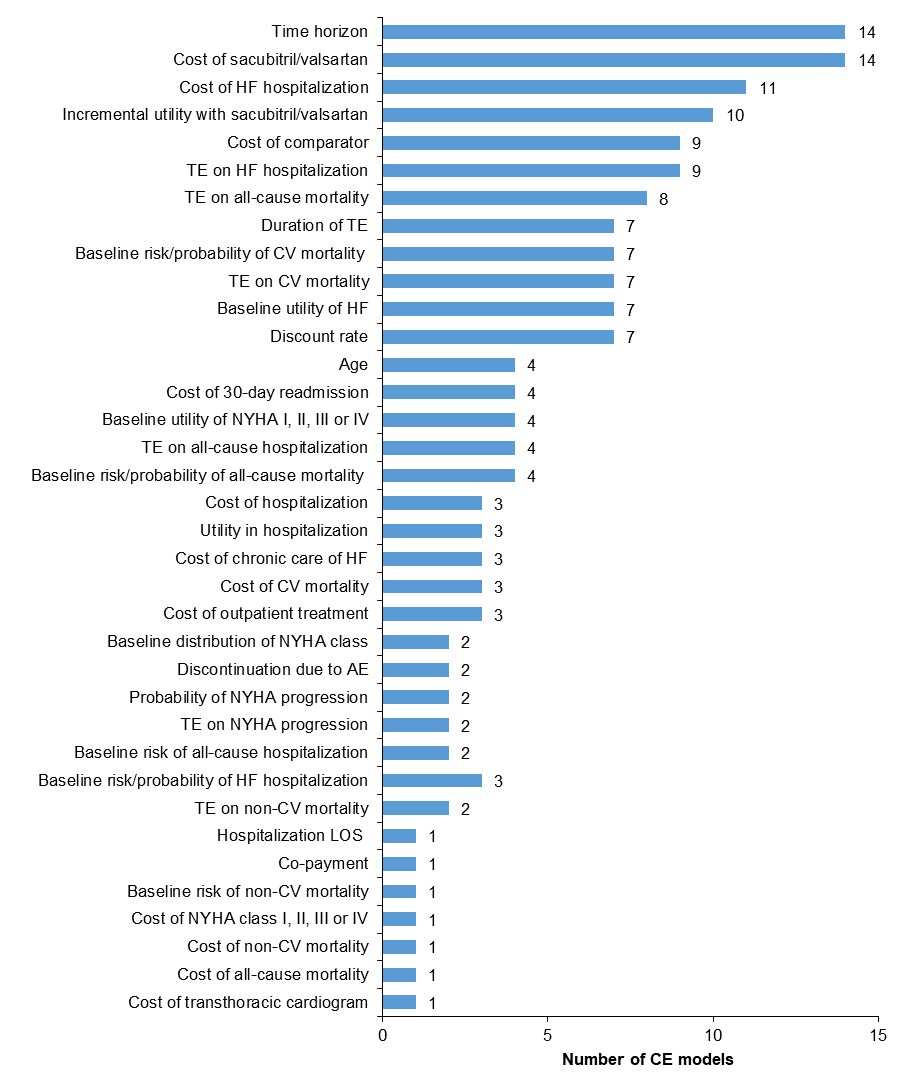


**(B) Markov and regression-based models**


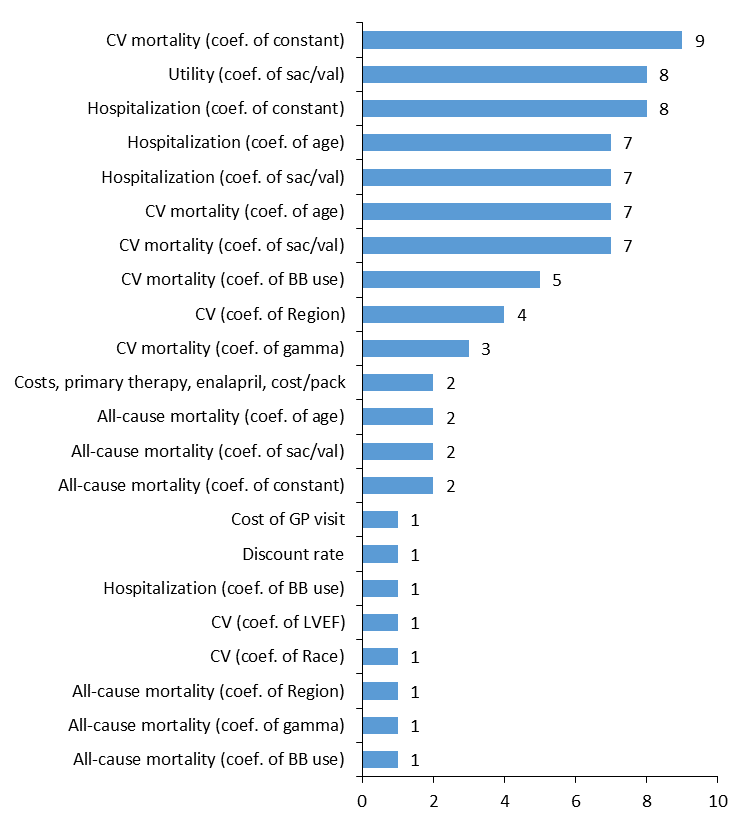


**Abbreviations**: AE, adverse event; BB, beta-blocker; CE, cost-effectiveness; CV, cardiovascular; GP, general practitioner; HF, heart failure; LOS, length of stay; LVEF, left ventricular ejection fraction; NYHA, New York Heart Association; sac/val, sacubitril/valsartan; TE, treatment effect.

Figure 2. Quality of included studies and HTA reports

Questions included in the checklist:

1. Was the research question stated? 2. Was the economic importance of the research question stated? 3. Was/were the viewpoint(s) of the analysis clearly stated and justified? 4. Was a rationale reported for the choice of the alternative programs or interventions compared? 5. Were the alternatives being compared clearly described? 6. Was the form of economic evaluation stated? 7. Was the choice of form of economic evaluation justified in relation to the questions addressed? 8. Was/were the source(s) of effectiveness estimates used stated? 9. Were details of the design and results of the effectiveness study given (if based on a single study)? 10. Were details of the methods of synthesis or meta-analysis of estimates given (if based on an overview of a number of effectiveness studies)? 11. Were the primary outcome measure(s) for the economic evaluation clearly stated? 12. Were the methods used to value health states and other benefits stated? 13. Were the details of the subjects from whom valuations were obtained given? 14. Were productivity changes (if included) reported separately? 15. Was the relevance of productivity changes to the study question discussed? 16. Were quantities of resources reported separately from their unit cost? 17. Were the methods for the estimation of quantities and unit costs described? 18. Were currency and price data recorded? 19. Were details of price adjustments for inflation or currency conversion given? 20. Were details of any model used given? 21. Was there a justification for the choice of model used and the key parameters on which it was based? 22. Was the time horizon of cost and benefits stated? 23. Was the discount rate stated? 24. Was the choice of rate justified? 25. Was an explanation given if cost or benefits were not discounted? 26. Were the details of statistical test(s) and confidence intervals given for stochastic data? 27. Was the approach to sensitivity analysis described? 28. Was the choice of variables for sensitivity analysis justified? 29. Were the ranges over which the parameters were varied stated? 30. Were relevant alternatives compared? (That is, were appropriate comparisons made when conducting the incremental analysis?) 31. Was an incremental analysis reported? 32. Were major outcomes presented in a disaggregated as well as aggregated form? 33. Was the answer to the study question given? 34. Did conclusions follow from the data reported? 35. Were conclusions accompanied by the appropriate caveats? 36. Were generalizability issues addressed?

**Abbreviations:** CADTH, Canadian Agency for Drugs and Technologies in Health; HTA, Health Technology Assessment; ICER, Institute for Clinical and Economic Review; NICE, National Institute for Health and Care Excellence; PBAC, Pharmaceutical Benefits Advisory Committee; SMC, Scottish Medicines Consortium.
